# A simple arthroscopic technique for treatment of displaced “hinged” type of posterior cruciate ligament avulsion fractures

**DOI:** 10.1186/s12891-022-05795-8

**Published:** 2022-09-03

**Authors:** Zheshu Xu, Yunlong Dong, Yu-e Feng, Peng Xie, Juyuan Gu, Kai Kang, Shijun Gao, Xiaozuo Zheng

**Affiliations:** 1 Department of Orthopedics, Third Hospital of Hebei Medical University, 139 Ziqiang Road, Shijiazhuang, 050051 Hebei China; 2 Key Laboratory of Biomechanics of Hebei Province, 139 Ziqiang Road, Shijiazhuang, 050051 Hebei China; 3 Department of Nuclear Medicine, Third Hospital of Hebei Medical University, 139 Ziqiang Road, Shijiazhuang, 050051 Hebei China

**Keywords:** Posterior cruciate ligament, Avulsion fracture, Tibia, Arthroscopy

## Abstract

**Background:**

The surgical technique for treatment of tibial avulsion fractures of the posterior cruciate ligament (PCL) remains challenging due to the deep-located lesion and the complexity of the anatomy. The purpose of this study was to report preliminary results of an arthroscopic technique in patients with the “hinged” type PCL tibial avulsion fractures.

**Methods:**

Twenty-eight patients with the displaced “hinged” fractures with elevation of the posterior aspect of the bony fragment were arthroscopically treated. The bony fragment was reducted and fixed with the sutures passing through only one single tibial tunnel. The clinical outcomes were assessed by Lysholm score, Tegner activity score, and the side-to-side differences of KT-1000 measurement. The reduction and union of the fracture were assessed by radiography of the knee.

**Results:**

Patients were followed up for a mean of 19 (12 to 24) months. There were no surgery-related complications, and all patients regained normal range of motion of the knees at the last follow-up. The Lysholm score significantly increased from preoperative 14.78 ± 8.23 to postoperative 96.96 ± 3.62 (*P* = 0.000). The Tegner score was 6.78 ± 1.35 pre-injury and 6.48 ± 1.20 at the last follow-up with no statistical difference (*P* = 0.688). The KT-1000 side-to-side differences significantly decreased from 8.26(SD 1.86; 6 to 12) pre-operatively to 0.91 (SD 0.85; 0 to 3) (*P* = 0.000). X-rays showed that satisfactory reduction and solid union was achieved in all patients.

**Conclusion:**

The arthroscopic suture fixation through single-tibial tunnel technique yielded good clinical and radiographic outcome for treatment of displaced “hinged” type of PCL avulsion fractures.

## Background

Avulsion fracture of the tibial insertion of the posterior cruciate ligament (PCL) is commonly occur among the young, and accounts for approximately 3–40% of all acute knee joint injuries [1]. It mainly results from motorcycle accidents and hyperflexion injuries of the knee in sport [2], and is associated with a high rate of functional impairment. Meyers-McKeever proposed a classification system for the cruciate ligament avulsion fractures, which divides them into Type I (minimally displaced avulsion), II (hinged avulsion), and III (completely detached avulsion) [3]. The “hinged” type fractures (type II) have elevation of the posterior aspect of the avulsed fragment, and have been treated non-operatively. While, a malunion fracture after non-operative treatment would cause loss of function of PCL, leading to knee instability, decreased sporting activity, and long-term degeneration [4]. Some authors even reported that knee instability could still be found even though the bony fragment had been surgically reduced, since the plastic changes of the cruciate ligament at the time of injury could not be avoided [5, 6]. Therefore, the displaced avulsion fracture of PCL should be anatomically reduced and fixed to restore the normal tension and function of the ligament [7]. Open reduction and fixation of the PCL avulsion fracture through a posterior approach is a common procedure, but the exposure of the fracture is difficult due to the deep-located lesion and the complexity of the anatomy [8, 9]. Arthroscopically assisted reduction and fixation of the PCL avulsion fractures is gradually preferred since it is a minimally invasive technique. Multiple fixation devices have been described, such as Kirschner wire [10], cannulated screw [11], and pullout sutures [12]. The suspensory suture-based fixation was introduced in recent years, and could provide firm fixation for early rehabilitation. The previous biomechanical studies confirmed that it was as reliable as screw fixation for this lesion in terms of initial fixation [13, 14]. As the arthroscopical suture fixation technique is technically demanding and there is still risk of injury to the popliteal vessels and nerves, it is more complicated to establish double or triple tunnels and to shuttle the sutures for fracture reduction and fixation, when compared with the single tibial tunnel technique [15]. Therefore, we describe an arthroscopic reduction and fixation technique with the sutures passing through only one single tibial tunnel for treatment of the “hinged” type PCL tibial avulsion fractures.

The purpose of this study was to report the clinical and radiologic results of this procedure in patients with displaced “hinged” type (Type II) PCL tibial avulsion fractures. Our hypothesis was that this procedure would be easy to perform, and effective in these patients.

## Methods

### Participants

This retrospective study was carried out with the approvement of the ethics committee of our institution. The diagnosis PCL tibial avulsion fracture was based on X-ray and the computed tomography (CT) scan. Magnetic resonance imaging (MRI) was used to detect whether meniscal or ligament injuries existed. Displacement of the avulsed fragment was determined in the sagittal plane on CT scan by measuring the distance from distal-most point of the bony fragment to the distal margin of the fracture bed. The inclusion criteria were as follows: (1) Patients with acute tibial avulsion fracture of PCL up to 3 weeks before surgery, (2) the displaced “hinged” type fractures (type II) with elevation of the posterior aspect of the bony fragment more than 3 mm, (3) the posterior knee instability of grade II or higher evaluated by the KT-1000 arthrometer [16], (4) skeletally mature patients older than 16 years, (5) no associated tibial plateau fracture or coexistent ligament injuries that needed surgery. The exclusion criteria were as follows:(1) Patients with tibial avulsion fracture of PCL more than 3 weeks before surgery, (2) the displaced “hinged” type fractures (type II) with elevation of the posterior aspect of the bony fragment ≤ 3 mm, (3) the posterior knee instability less than grade II evaluated by the KT-1000 arthrometer, (4) skeletally immature patients, (5) Fractures around the tibial plateau other than tibial avulsion fracture of PCL, (6) Combined anterior cruciate ligament injuries, collateral ligament injuries, or severe multiple ligament injuries, (7) Severe neurovascular injury associated with the knee. Patients were excluded if they did not meet the inclusion criteria, or did not finish at least 12 months follow-up.

A total of 23 (10 male and 13 female patients) patients who met the inclusion criteria, treated between June 2017 and February 2020, were enrolled in this study. The mean age at the time of surgery was 43 years (range, 22 to 59 years). The time from injury to surgery was 10 days (range, 3 to 21 days). The injury mechanisms included 13 road traffic accidents, 1 sport injuries, and 9 fall injuries. All the patients were treated with the same surgical technique. The posterior drawer test and KT-1000 arthrometer examination (MEDmetric, San Diego, California) were performed under anaesthesia before surgery. All patients were followed up for a mean of 19 months (range 12–28 months).

### Surgical procedure

**Fig. 1:**
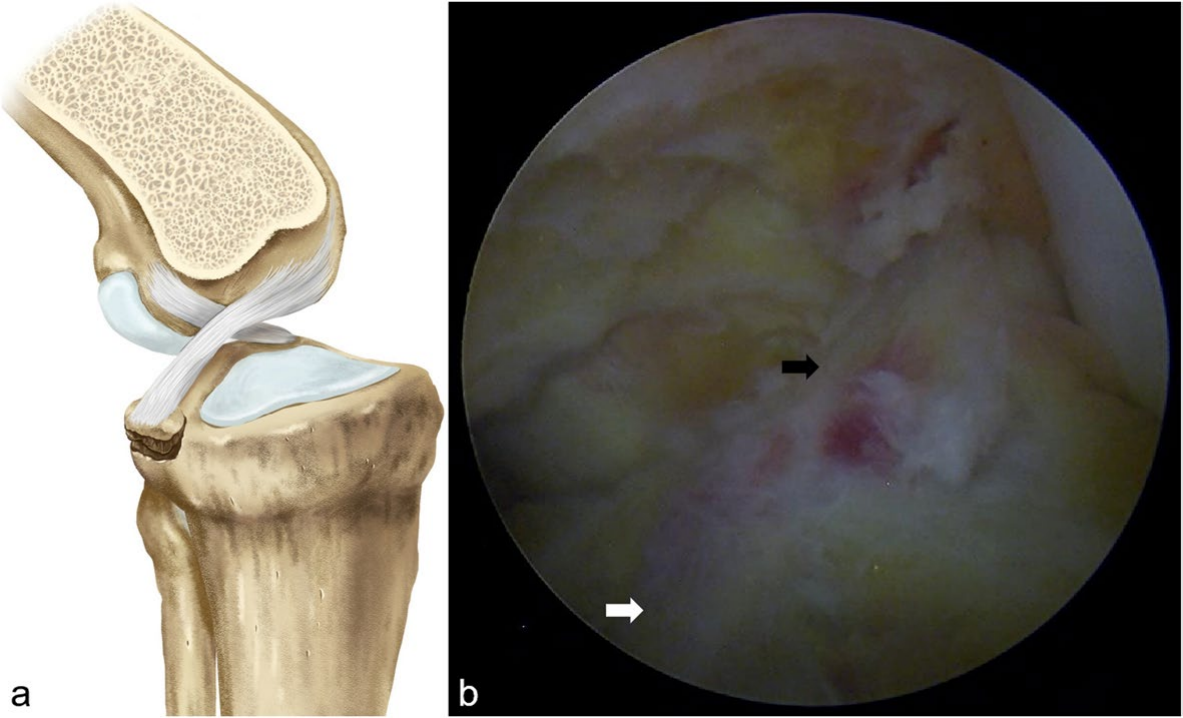
The displaced “hinged” type (Type II) PCL tibial avulsion fracture. **a** Illustration showing the displaced “hinged” type fracture with elevation of the posterior aspect of the bony fragment (black arrow). **b** Arthroscopic view showing part of the posterior septum was removed, and the PCL (black arrow) and the avulsed tibial fragment (white arrow) were ascertained

**Fig. 2:**
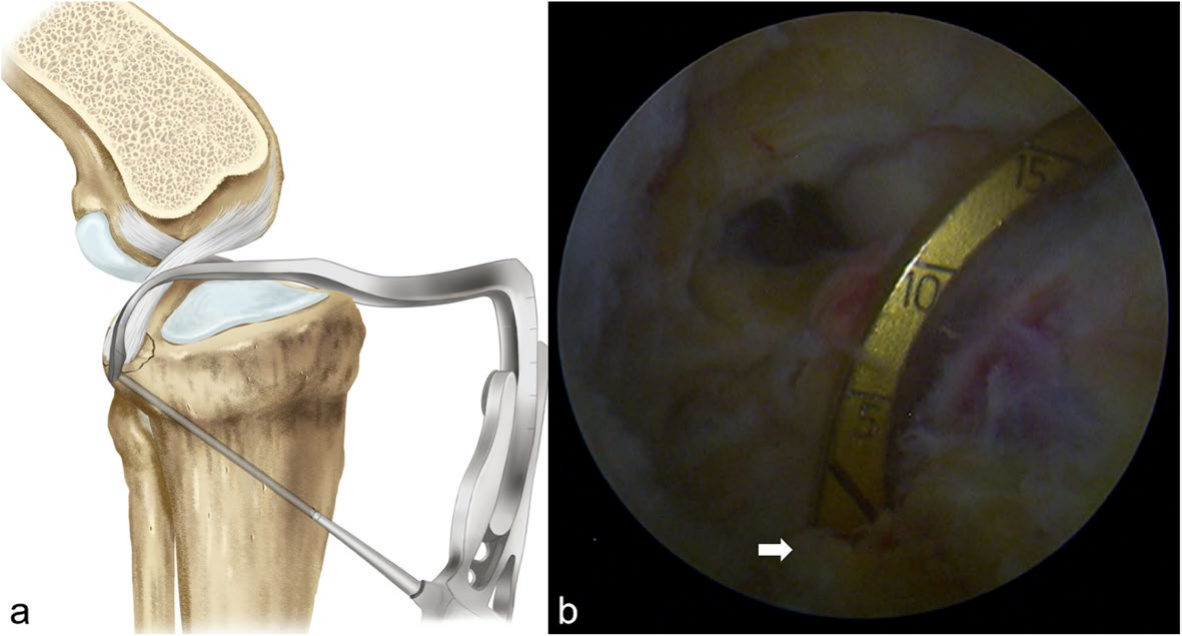
Bony fragment reduction with the tip of the guide. **a** Illustration showing the bony fragment temporarily reduced and fixed with the tip of the guide, and a guide pin was drilled from the anterior tibial cortex into the avulsed fragment. **b** Arthroscopic view showing the avulsion fracture fragment was reduced with the the tip of the guide (white arrow)

**Fig. 3:**
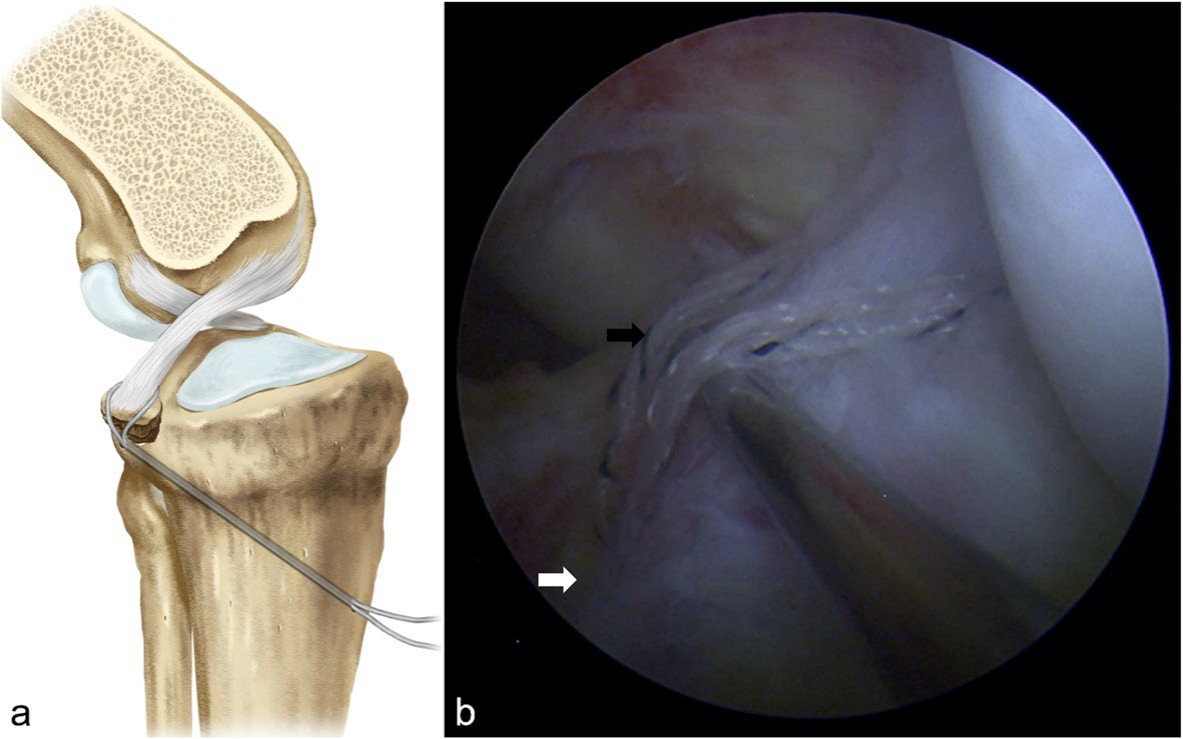
PCL base wrapped with FiberTape suture. **a** Illustration showing the PCL base was wrapped the from anterior to posterior using a FiberTape suture. **b** Arthroscopic view showing the inner opening of the tunnel located at the bottom of the tibial fracture bed (white arrow), and the PCL was wrapped with FiberTape suture (black arrow).

**Fig. 4:**
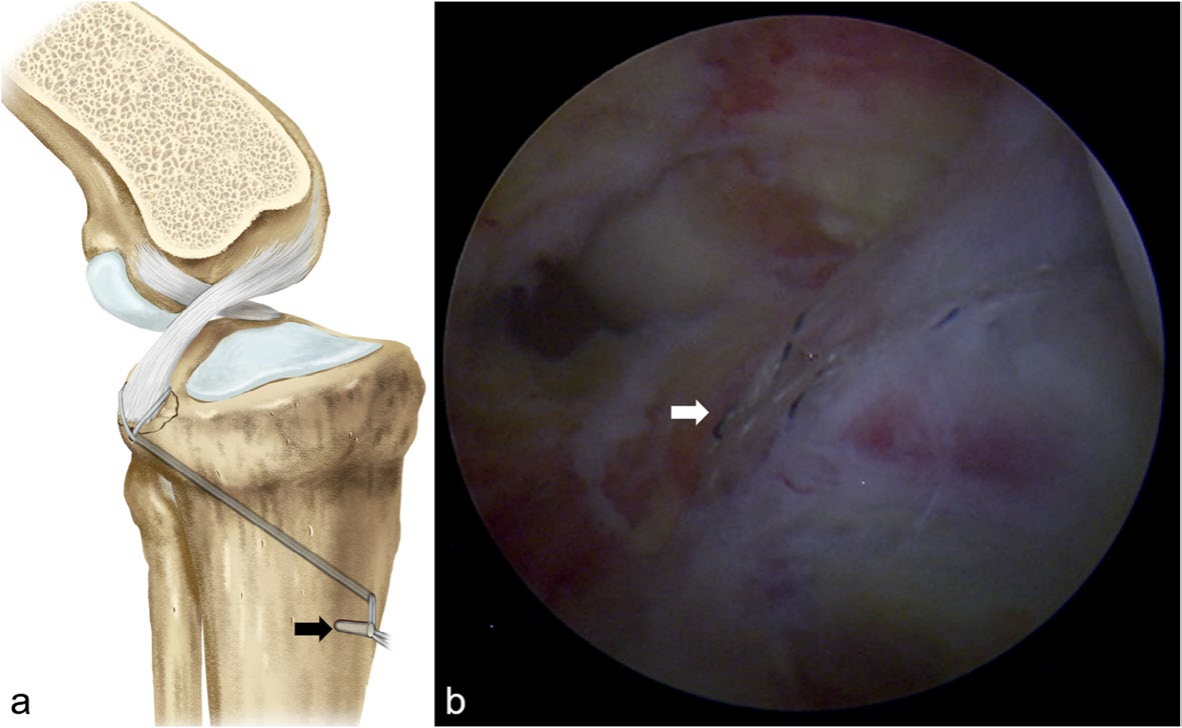
Reduction and fixation of avulsed fracture. **a** Illustration showing the reduction of avulsion fracture was achieved with the tightened suture and fixed with the Knotless device (black arrow). **b** Arthroscopic view showing the tightened suture (white arrow), and the tension of PCL was regained

Spinal or general anesthesia was administered. Patients were positioned supine and a thigh tourniquet was used. The diagnostic arthroscopy was routinely performed and the concomitant lesions were treated through anteromedial (AM) and anterolateral (AL) portals. The high and low posteromedial (PM) portals were then established under direct visualization with the arthroscope placed from the AL portal, through the intercondylar notch, into the posteromedial compartment. The arthroscope was then placed through the high PM portal, and the shaver was placed through the low PM portal. Part of the posterior septum was removed until the margins of the avulsed fragment and the tibial bed were ascertained (Fig. 1). After completion of the initial diagnostic arthroscopy, a 1.5 cm long incision was made 2 to 3 cm medial to the tibial tubercle. With the arthroscope placed in high PM portal, the PCL tibia guide (Smith&Nephew Endoscopy, Andover, MA) was introduced into the posterior compartment through the AM portal. The bony fragment was reduced and fixed temporarily with the tip of the guide placed at lower part or the bottom of the tibial fracture bed (Fig. 2). A 2 mm guide pin was drilled from the anterior tibial cortex into the avulsed fragment, and then the guide pin was over-drilled with a 4.5-mm cannulated reamer. A FiberTape suture (Arthrex, Naples, FL) was introduced through the AL portal, and wrapped the PCL base from anterior to posterior. One end of the suture passed between the PCL and the medial femoral condyle, and the other end of the suture passed between the anterior cruciate ligament (ACL) and the PCL (Fig. 3). Attention should be paid not to wrap the ACL and meniscal-femoral ligaments (Humphry’s or Wrisberg’s ligament). To avoid this wrong wrapping, the arthroscope could be changed to the AM portal for direct observation, and to make sure that the sutures pass into the posterior compartment through the medial side of the ACL and the underside of the meniscal-femoral ligaments. After completion of the suturing process, the ends of the sutures were grasped and pulled out of the tibial tunnel. A sliding knot was placed at the base of the PCL through the tibial tunnel, followed by 2 to 3 half-hitches to secure the knot. The bony fragment was reduced to the anatomical position using a probe through the PM portal. Finally, the two ends of the suture were tightened to achieve reduction, and fixed with the Knotless device (Swivelock, Arthrex, Naples, FL), which located distally to the outer opening of the tibial tunnel (Fig. 4).

### Rehabilitation protocol

The knee was immobilized with a long hinge brace postoperatively for 12 weeks. Isometric quadriceps training was started immediately after surgery. Protected passive range of motion (ROM) exercise was started 2 weeks after the surgery, with the goal of reaching normal ROM within 8 weeks. Partial weight bearing using crutches is permitted from the fourth postoperative week, and full weight bearing was started at 8 weeks. The brace was removed and patients are encouraged to increase activity gradually 3 months after surgery. Patients were allowed to do sports activities at 6 months after surgery.

### Clinical evaluation

All the patients underwent clinical assessments preoperatively and postoperatively, and were routinely followed up. Knee function was assessed by Lysholm score and Tegner activity score. Knee stability was evaluated by the side-to-side differences of KT-1000 arthrometer (MEDmetric, San Diego, CA, USA). All patients underwent anteroposterior and lateral radiography of the knee immediately after surgery, at 3 months, and at final follow-up.

### Statistical analysis

All statistical analyses were performed using SPSS software version 21.0 (version 19.0; SPSS Inc., Chicago, IL). The t-test was used to compare the pre-injury and post-operative Tegner scores, and the pre- and post-operative Lysholm score and KT1000 side-to-side differences. The level of significance was set at *P* < 0.05.

## Results

The mean operative time was 56 (range 45–75) minutes. There were no neurovascular injuries, infection, arthrofibrosis, or other surgery-related complications. There was no hardware-related complication.

Patients were followed up for a mean of 19 (12 to 24) months. All patients regained normal ROM with no extension or flexion deficit, and had returned to their daily life and work after surgery. The Lysholm score significantly increased from preoperative 14.78(SD 8.23; 5 to 30) to postoperative 96.96 (SD 3.62; 89 to 100) at the final follow-up (*P* = 0.000). The sports activity evaluation of Tegner score was 6.78(SD 1.35; 4 to 9) pre-injury and 6.48(SD 1.20; 4 to 9) at the final follow-up, with no statistical difference(*P* = 0.688). The KT-1000 side-to-side differences significantly decreased from 8.26(SD 1.86; 6 to 12) pre-operatively to 0.91 (SD 0.85; 0 to 3) at final follow-up (*P* = 0.000).

**Fig. 5:**
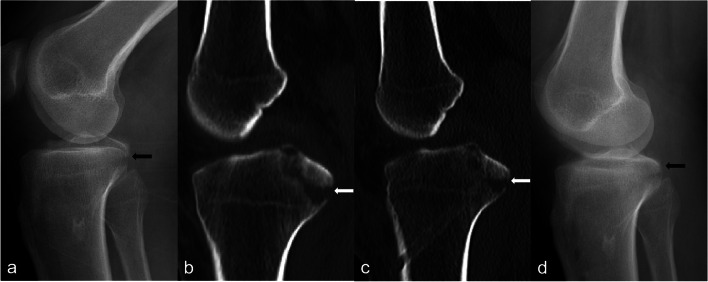
Radiographic examination at preoperative and postoperative. **a**-**b **Preoperative X-ray (black arrow) and CT Scans (white arrow) show the elevated bony frament of Type II PCL Tibial Avulsion Fractures. **c** Immediate postoperative CT scan show the satisfactory reduction of the fracture(white arrow). **d** Postoperative X-ray at final follow-up showed that reduction of the fracture was satisfactory, and solid union was achieved (black arrow)

Radiographic examination showed that the fracture has reached anatomical reduction, and solid union achieved in all patients at final follow-up (Fig. 5). No displacement or nonunion of the fracture was found.

## Discussion

The unstable displaced “hinged”type PCL tibial avulsion fractures could be satisfactorily reduced through single tibial tunnel, and solidly fixed by the simple sutures. This technique is simple, reproducible, and showed good clinical and radiological outcomes.

**Fig. 6:**
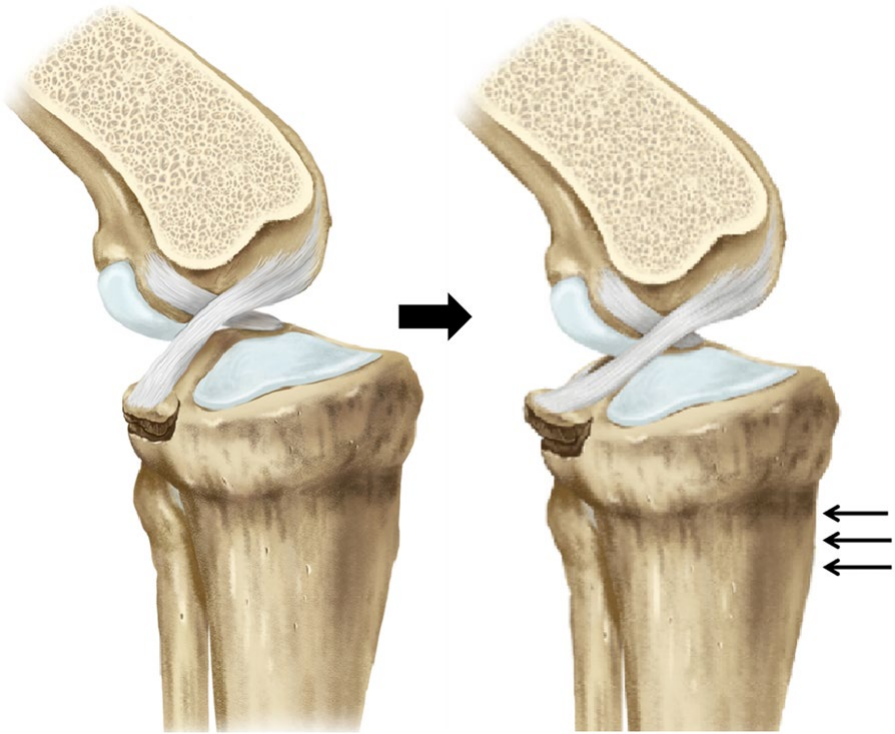
Illustration showing the displacement of the fracture would aggravate with posterior stress (three black line arrows)

The PCL plays an important role in knee joint stabilization. Avulsion fractures constitute a part of the PCL injuries [17]. Treatment is determined by the type of avulsion fracture. The general consensus is that the minimally displaced lesions (type I) are typically treated conservatively, and surgery is recommended for completely displaced fractures (type III). While, there is still no consensus on how to treat type II lesions(displaced “hinged” type fractures), with being treated either conservatively or surgically. We do recommend surgical reduction and rigid fixation for the “hinged” type fractures because of the following reasons. First, we believe that the “hinged” type fractures is a type unstable fracture, since the displacement would be aggravated when the knee under posterior stress as the Fig. 6 shown. The non-operative fixation with the external brace is an indirect fixation method. It is not rigid enough to stabilize the fracture, but was associated with a high risk of knee stiffness. Second, the non-operative treatment strategy has little effect on reduction for the displaced fragments. Proper union will not occur if the anatomical reduction is not achieved. That would result in loss function of PCL, leading to knee instability and delayed arthritis [18]. Therefore, surgical treatment of displaced tibial avulsion fractures of the PCL is necessary to achieve anatomic reduction and rigid fixation, restore proper tension to the PCL and achieve knee stability. In our study, we enrolled the type II fractures with elevation of the posterior aspect of the bony fragment more than 3 mm, and the posterior knee instability of grade II or higher. On the basis of current results, we found that the avulsed fragment and bony union were achieved in all the patients. The knee stability and function was satisfactory at final follow-up.

The advantages of arthroscopic treatment for the PCL avulsion fractures include less surgical invasion, less danger to the popliteal neurovascular structures, and concomitant lesions could be treated. Our arthroscopic technique is similar to the previous methods but with some modifications [12, 19]. We used double PM portals, which provide clear visualisation of the tibial bed, and satisfactory access for debridement and sutures shuttling. The suture implant has become the most widely used implant for fixation of cruciate ligament avulsion fractures, since it is versatile and could be used irrespective of the size of the bony fragment. For suture fixation technique, one or more tibial tunnels are required for the passage and fixation of sutures. The most commonly used tunnels are single [15], double [12], or triple shaped [20]. We do agree with Gui et al. [15] and Sabat et al. [7] that single tibial tunnel method is simple in tunnel establishing and the sutures shuttling in the narrow posterior knee compartment when compared with the double or triple tunnel techniques. In our experience, the displaced lower part of the PCL tibial insertion could be easily reducted using the tip of PCL tibia guide, and the fragment could be maintained in the position with the sutures passing only one tibial tunnel. The key point is that the inner opening of the tunnel should be located placed at lower part or the bottom of the tibial fracture bed. We used the knotless device for fixation of the ends of the sutures. It is not a metal implant, and no further removal surgery was need. The disadvantage of the single tunnel technique could be the suture slippage. To prevent this from happening, it is better to make a knot at the base of the PCL after suturing process, and keep tension of the suture during the fixation process. Our arthroscopic reduction and fixation technique showed that the posterior knee stability was achieved immediately after fixation. There was no radiographic displacement of fragment or hardware complication was noted during the follow-up period.

### Limitation

There were several limitations in our study. First, this study is retrospective design with small number of patients included. We also did not set a control group with non-operative treatment strategy. These are because the incidence of PCL tibial avulsion fractures is relatively low. Second, the postoperative CT scan, which is more reliable than X-ray for evaluating the bony healing, was not routinely performed.

## Conclusion

The arthroscopic suture fixation through single-tibial tunnel technique yielded good clinical and radiographic outcome for treatment of displaced “hinged” type of PCL avulsion fractures.

## Data Availability

The datasets used and/or analyzed during the current study are available from the corresponding author on reasonable request.

## Abbreviations

PCL: Posterior cruciate ligament
CT: Computed tomography
MRI: Magnetic resonance imaging
AM: Anteromedial
AL: Anterolateral
PM: Posteromedial
AL: Anterolateral
ROM: Range-of-motion

## References

[1] Kim YM, Lee CA, Matava MJ (2011). Clinical results of arthroscopic single-bundle transtibial posterior cruciate ligament reconstruction : a systematic review. Am J Sports Med.

[2] Katsman A, Strauss EJ, Campbell KA, Alaia MJ (2018). Posterior cruciate ligament avulsion fractures. Curr Rev Musculoskelet Med.

[3] Griffith JF, Antonio GE, Tong CW, Ming CK (2004). Cruciate ligament avulsion fractures. Arthroscopy.

[4] Van de Velde SK, Bingham JT, Gill TJ, Li G. Analysis of tibiofemoral cartilage deformation in the posterior cruciate ligament-deficient knee.; 2009:167–75. 10.2106/JBJS.H.00177 PMC2663325 19122092

[5] Inoue M, Yasuda K, Kondo E, Saito K, Ishibe M (2004). Primary repair of posterior cruciate ligament avulsion fracture: the effect of occult injury in the m idsubstance on postoperative instability. Am J Sports Med.

[6] James H, Lubowitz WS (2005). Arthroscopic treatment of tibial plateau fractures: intercondylar eminence avulsion fractures. Arthroscopy.

[7] Sabat D, Jain A, Kumar V (2016). Displaced Posterior Cruciate Ligament Avulsion Fractures: A Retrospective Comparative Study Between O pen Posterior Approach and Arthroscopic Single-Tunnel Suture Fixation. Arthroscopy.

[8] Trickey EL. Injuries to the posterior cruciate ligament: diagnosis and treatment of early injuries and reconstruc tion of late instability. Clin Orthop Relat Res. 1980147):76–81. 7371320

[9] Zhao J, He Y, Wang J (2006). Arthroscopic treatment of acute tibial avulsion fracture of the posterior cruciate ligament with sutu re fixation technique through Y-shaped bone tunnels. Arthroscopy.

[10] Deehan DJ, Pinczewski LA (2001). Arthroscopic reattachment of an avulsion fracture of the tibial insertion of the posterior cruciate l igament. Arthroscopy.

[11] Shino K, Nakata K, Mae T, Yamada Y, Shiozaki Y, Toritsuka Y. Arthroscopic fixation of tibial bony avulsion of the posterior cruciate ligament. Arthroscopy: The Journal of Arthroscopic & Related Surgery. 2003;19(2):1–5. 10.1053/jars.2003.50062 12579141

[12] Madi SS, Pandey V, Reddy B, Acharya K (2021). Clinical and Radiological Outcomes Following Arthroscopic Dual Tibial Tunnel Double Sutures Knot-bump Fixation Technique for Acute Displaced Posterior Cruciate Ligament Avulsion Fractures. ARCH BONE JT SURG-AB.

[13] Sasaki SU, E Albuquerque RFDM, Amatuzzi MM, Pereira CAM. Open screw fixation versus arthroscopic suture fixation of tibial posterior cruciate ligament avulsion injuries: a mechanical comparison. Arthroscopy: The Journal of Arthroscopic & Related Surgery. 2007;23(11):1226–30. 10.1016/j.arthro.2007.06.012 17986411

[14] Domnick C, Kösters C, Franke F, Raschke MJ, Petersen W, Fink C,et al. Biomechanical properties of different fixation techniques for posterior cruciate ligament avulsion fractures. Arthroscopy: The Journal of Arthroscopic & Related Surgery. 2016;32(6):1065–71. 10.1016/j.arthro.2015.10.013 26775734

[15] Gui J, Wang L, Jiang Y, Wang Q, Yu Z, Gu Q. Single-tunnel suture fixation of posterior cruciate ligament avulsion fracture. Arthroscopy: The Journal of Arthroscopic & Related Surgery. 2009;25(1):78–85. 10.1016/j.arthro.2008.08.011 19111222

[16] Keller PM, Shelbourne KD, McCarroll JR, Rettig AC (1993). Nonoperatively treated isolated posterior cruciate ligament injuries. Am J Sports Med.

[17] Yang CK, Wu CD, Chih CJ, Wei KY, Su CC, Tsuang YH (2003). Surgical treatment of avulsion fracture of the posterior cruciate ligament and postoperative manageme nt. J Trauma.

[18] Cosgarea AJ, Jay PR (2001). Posterior cruciate ligament injuries: evaluation and management. J Am Acad Orthop Surg.

[19] Zheng W, Hou W, Zhang Z, Li P, Zhou B, Li H,et al. Results of Arthroscopic Treatment of Acute Posterior Cruciate Ligament Avulsion Fractures With Suspensory Fixation.; 2021:1872–80. 10.1016/j.arthro.2021.01.044 33539975

[20] Rhee SJ, Jang JH, Choi YY, Suh JT (2019). Arthroscopic reduction of posterior cruciate ligament tibial avulsion fracture using two cross-linked pull-out sutures: A surgical technique and case series. Injury.

